# Transcatheter edge-to-edge repair with DragonFly^TM^ system and ‘stepless self-locking’ strategy in a patient with mixed mitral regurgitation: a case report

**DOI:** 10.1093/ehjcr/ytag435

**Published:** 2026-06-20

**Authors:** Xinping Lin, Yuming Wang, Boren Tan, Jian’an Wang, Xianbao Liu

**Affiliations:** Department of Cardiology, The Second Affiliated Hospital, School of Medicine, Zhejiang University, Jiefang Road 88, Hangzhou 310000, China; State Key Laboratory of Transvascular Implantation Devices, Qidi Road 456, Hangzhou 310000, China; Heart Regeneration and Repair Key Laboratory of Zhejiang Province, Jiefang Road 88, Hangzhou 310000, China; Transvascular Implantation Devices Research Institute, Qidi Road 456, Hangzhou 310000, China; Department of Cardiology, The Second Affiliated Hospital, School of Medicine, Zhejiang University, Jiefang Road 88, Hangzhou 310000, China; State Key Laboratory of Transvascular Implantation Devices, Qidi Road 456, Hangzhou 310000, China; Heart Regeneration and Repair Key Laboratory of Zhejiang Province, Jiefang Road 88, Hangzhou 310000, China; Transvascular Implantation Devices Research Institute, Qidi Road 456, Hangzhou 310000, China; Department of Cardiology, The Second Affiliated Hospital, School of Medicine, Zhejiang University, Jiefang Road 88, Hangzhou 310000, China; State Key Laboratory of Transvascular Implantation Devices, Qidi Road 456, Hangzhou 310000, China; Heart Regeneration and Repair Key Laboratory of Zhejiang Province, Jiefang Road 88, Hangzhou 310000, China; Transvascular Implantation Devices Research Institute, Qidi Road 456, Hangzhou 310000, China; Department of Cardiology, The Second Affiliated Hospital, School of Medicine, Zhejiang University, Jiefang Road 88, Hangzhou 310000, China; State Key Laboratory of Transvascular Implantation Devices, Qidi Road 456, Hangzhou 310000, China; Heart Regeneration and Repair Key Laboratory of Zhejiang Province, Jiefang Road 88, Hangzhou 310000, China; Transvascular Implantation Devices Research Institute, Qidi Road 456, Hangzhou 310000, China; Binjiang Institute of Zhejiang University, Jucai Road 239, Hangzhou 310000, China; Department of Cardiology, The Second Affiliated Hospital, School of Medicine, Zhejiang University, Jiefang Road 88, Hangzhou 310000, China; State Key Laboratory of Transvascular Implantation Devices, Qidi Road 456, Hangzhou 310000, China; Heart Regeneration and Repair Key Laboratory of Zhejiang Province, Jiefang Road 88, Hangzhou 310000, China; Transvascular Implantation Devices Research Institute, Qidi Road 456, Hangzhou 310000, China; Binjiang Institute of Zhejiang University, Jucai Road 239, Hangzhou 310000, China

**Keywords:** Stepless self-locking, DragonFly^TM^, Transcatheter edge-to-edge repair, Mitral regurgitation, Case report

## Abstract

**Background:**

Transcatheter edge-to-edge repair (TEER) has emerged as an alternative treatment for severe mitral regurgitation (MR). However, complex anatomical features of mitral valve pose significant technical and procedural challenges for TEER. In patients whose effective regurgitation reduction requires multiple clips, a small mitral valve area (MVA) limits TEER by increasing the risk of post-procedural elevated transvalvular gradients and iatrogenic stenosis.

**Case summary:**

A 90-year-old man presented with progressive tightness of breath that remained refractory to drug-based medical therapy. Echocardiography revealed severe mixed mitral regurgitation characterized by C1 segment prolapse and atrial functional mitral regurgitation with a small MVA of 4.09 cm^2^. Given his advanced age and prohibitive surgical risk (Society of Thoracic Surgeons [STS] score for repair: 10.49%), the multidisciplinary Heart Team elected to perform TEER using the DragonFly^TM^ system. The procedure was successfully performed under transoesophageal echocardiographic (TEE) guidance with the sequential implantation of two clips. The second clip utilized a ‘Stepless self-locking’ strategy to carefully balance the reduction of regurgitation against the transmitral pressure gradient elevation. Regurgitation was reduced from grade 4 + to 1 + with a final mean gradient of 4 mmHg and no evidence of significant mitral stenosis. The patient experienced a marked improvement in functional status during the follow-up period.

**Discussion:**

This case highlights the importance of individualized anatomical assessment and tailored device selection in managing patients with MR and complex, challenging structure. Precise clip sizing and stepwise deployment strategies can successfully balance MR reduction while preserving MVA. Such tailored approaches are essential to prevent iatrogenic complications and optimize clinical outcomes in high-risk populations.

Learning points

- Small mitral valve area poses a critical challenge for TEER, particularly with complex lesions and when multiple clips are required.
- A ‘Stepless self-locking’ strategy enables precise control of clip closure to balance regurgitation reduction and transmitral gradient.
- Tailored device selection and stepwise deployment are essential for optimizing outcomes in complex mitral regurgitation.

## Introduction

Transcatheter edge-to-edge valve repair has become an established therapeutic alternative for patients with symptomatic functional or degenerative mitral regurgitation who are at prohibitive surgical risk.^1–3^ However, the clinical management of mixed mitral regurgitation (MR), where primary degenerative prolapse and secondary functional mechanisms coexist, presents a significantly higher level of complexity.^4,5^ This complexity is further amplified in patients with a small mitral valve area (MVA), where the implantation of multiple clips to achieve adequate regurgitation reduction may result in excessive narrowing of the valve orifice. Such interventions carry a substantial risk of iatrogenic mitral stenosis, a complication associated with adverse clinical outcomes and limited symptomatic improvement.^6^ Here, we report the successful treatment of an elderly, high-risk patient with severe mixed MR and a small MVA using the DragonFly^TM^ system^7^ in combination with a ‘Stepless self-locking’ strategy,^8^ enabling a tailored balance between effective regurgitation reduction and preservation of valve area.

## Summary figure

**Figure:**
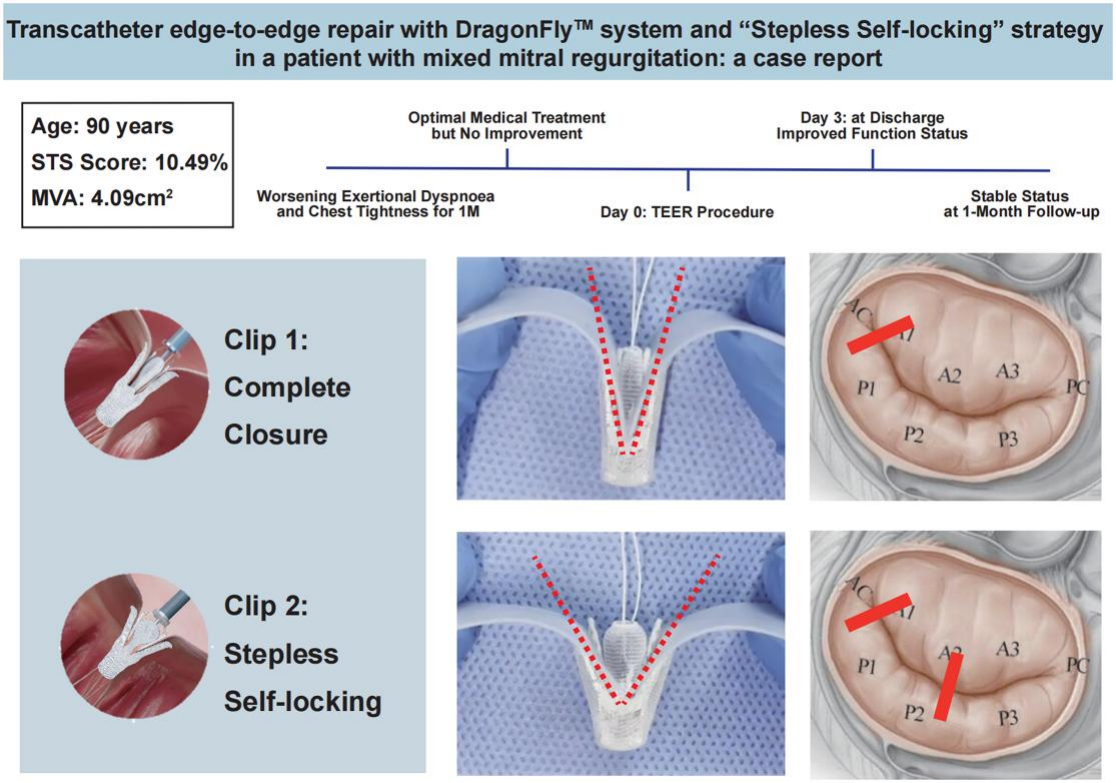


## Case presentation

A 90-year-old male was admitted with a 1-month history of worsening exertional dyspnoea and chest tightness. His medical history included atrial fibrillation, complete atrial-ventricular block, and permanent pacemaker implantation. Physical examination revealed a grade 4/6 murmur at the apex, accompanied by bilateral pulmonary crackles. A transthoracic echocardiogram performed at clinic suggested severe mixed mitral valve regurgitation. Optimal medical therapy was initiated 1 month before this admission, including edoxaban 30 mg daily, dapagliflozin 10 mg daily, furosemide 20 mg daily, and spironolactone 20 mg daily, but symptoms remained refractory. Therefore, he was admitted for comprehensive assessment and management.

**Figure 1:**
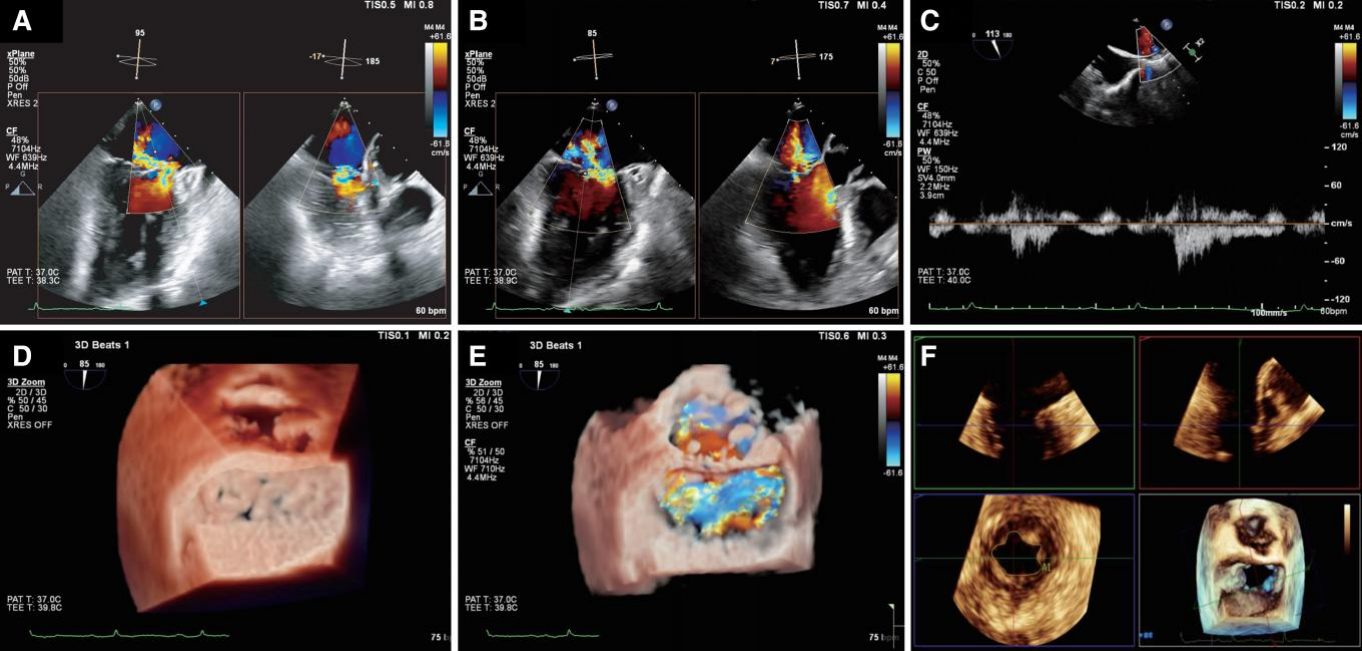
Preprocedural transoesophageal echocardiographic assessment mitral valve (*A*) TEE showing prolapse of the C1 segment and mitral regurgitation. (*B*) TEE showing central mitral regurgitation. (*C*) TEE showing the pulmonary vein flow reversal. (*D*) Three-dimensional TEE showing the location of leaflet prolapse. (*E*) Three-dimensional TEE showing two distinct regurgitant jets. (*F*) Assessment of mitral valve area. TEE, transoesophageal echocardiography.

Upon admission, the patient was classified as New York Heart Association (NYHA) functional Class IV (*Table 1*) and was unable to perform a 6-min walk test due to severe exertional intolerance. Transoesophageal echocardiographic (TEE) revealed severe mixed mitral regurgitation characterized by degenerative prolapse of the C1 segment in combination with atrial functional regurgitation in the A2/P2 region (*Figure 1A–E*). The prolapsed segment measured 1.1 cm in width with a 0.4 cm cleft-like gap. Leaflet lengths were measured at 1.6 cm for the anterior and 1.0 cm for the posterior leaflets. The left atrium was significantly dilated with a transverse diameter of 4.11 cm (*Figure 1F*), and moderate tricuspid regurgitation was present. Given the patient’s advanced age and high surgical risk, the Heart Team opted for transcatheter mitral valve edge-to-edge repair.

**Table 1:** Baseline, discharge and 1-month follow-up characteristics, echocardiographic and clinical parameters

	Baseline	Discharge	1-month follow-up
Mitral regurgitation	4+	1+	1+
Mitral valve area^a^, cm^2^	4.03	/	/
Mean transvalvular gradient, mmHg	1	4	4
Tricuspid regurgitation	2+	2+	1+
IVSd, cm	0.85	1.03	0.94
LVIDd, cm	4.66	4.48	4.20
LVPWd, cm	0.86	0.81	1.01
LVEF, %	65.5	60.4	66.4
LAV, mL	86.2	57.8	54.4
LAVi, mL/m^2^	56.0	37.5	34.0
PASP, mmHg	31	32	28
NYHA class	III	II	I

IVSd, interventricular septum diastole; LAV, left atrial volume; LAVi, left atrial volume index; NYHA, New York Heart Association; LVEF, left ventricular ejection fraction; LVIDd, left ventricular internal dimension diastole; LVPWd, left ventricular posterior wall dimension; PASP, pulmonary artery systolic pressure

^a^Mitral valve area measurement was not feasible because of acoustic shadowing and imaging interference related to the implanted clips.

**Figure 2:**
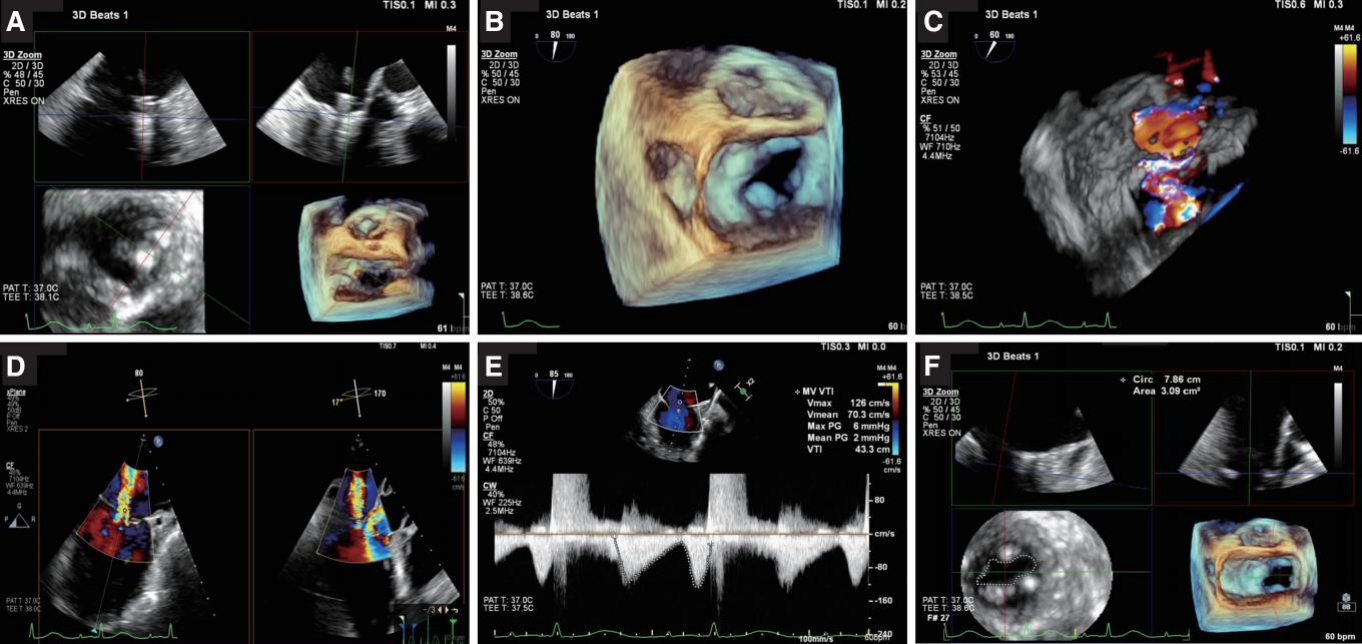
First clip implantation under transoesophageal echocardiographic guidance (*A–B*) deployment of the first clip at the lateral commissural region. (*C–D*) Residual central mitral regurgitation after first clip implantation. (*E*) Mean transmitral pressure gradient after first clip implantation. (*F*) Mitral valve area after first clip implantation.

The procedure was performed under general anaesthesia with TEE and digital subtraction angiography (DSA) guidance. Venous access was obtained via the right femoral vein, and a transseptal puncture was executed at a height of 4.72 cm. A short, wide clip (SW0609) was first navigated to the lateral commissure to address the primary prolapse. Under TEE guidance, the clip was oriented perpendicularly to the coaptation line and successfully deployed after confirming adequate leaflet capture (*Figure 2A* and *B*). The mitral regurgitation was reduced to grade 3 + with a mean gradient of 2 mmHg (*Figure 2C–E*). However, the significant atrial functional regurgitation persisted but with the MVA of only 3.09 cm^2^ (*Figure 2F*).

**Figure 3:**
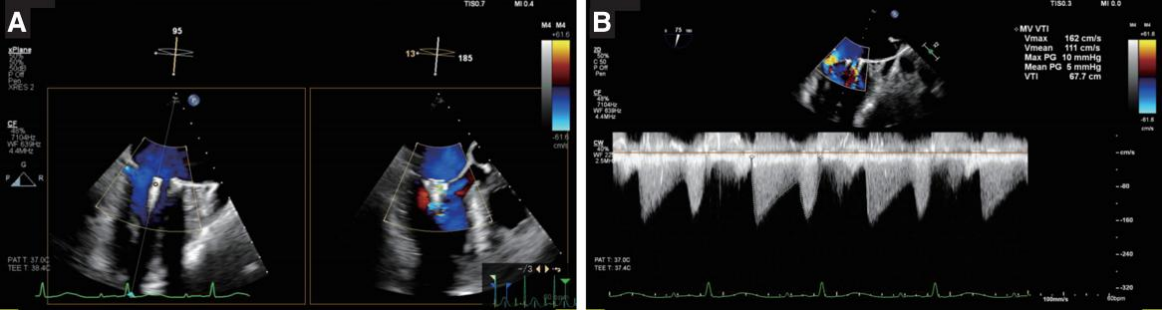
Second clip implantation and optimization strategy (*A–B*) ultimate incomplete deployment of a short, narrow clip.

**Figure 4:**
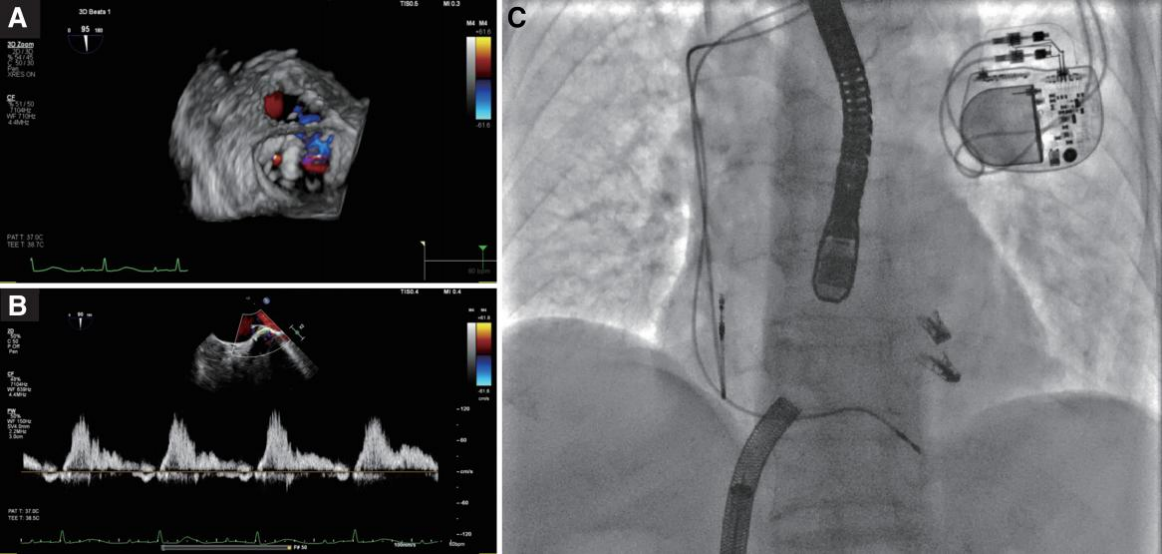
Postprocedural assessment after two clips implantation (*A*) trace residual mitral regurgitation after procedure. (*B*) Reduced pulmonary vein flow reversal. (*C*) DSA showing the relative position and orientation of the two clips, with the inferior clip (the second) deployed at a large opening angle. TEE, transoesophageal echocardiography.

Then, a second short, wide clip was attempted at the A2/P2 region, but the complete deployment resulted in a mean transmitral gradient of 8 mmHg (see Supplementary material online, *Figure S1A* and *B*), and the gradient remained 7 mmHg when incomplete deployment (see Supplementary material online, *Figure S1C and D*). The clip was subsequently retracted and replaced with a short, narrow clip (SN0409). However, the complete deployment still resulted in a mean gradient of 7 mmHg (see Supplementary material online, *Figure S1E* and *F*). Subsequently, our team attempted to utilize the DragonFly^TM^ system with its proprietary ‘Stepless self-Locking’ strategy, which is uniquely designed to maintain stable interaction between the clip and leaflets without requiring complete clip closure. The second clip was then employed with a large opening angle, but getting stable coaptation with trace residual regurgitation and a final mean transmitral gradient of 5 mmHg (*Figure 3A* and *B*; *Figure 4*).

The patient was successfully discharged on the third postoperative day in stable condition. At 1-month follow-up, he showed marked clinical improvement on functional status. Transthoracic echocardiography confirmed stable clip positions and trace residual regurgitation. The mean transmitral pressure gradient was 4 mmHg.

## Discussion

In recent years, transcatheter edge-to-edge repair has become an established therapy for mitral regurgitation. However, procedural success is highly dependent on suitable anatomical characteristics of the mitral valve.^9^ In the present case, complex mitral valve pathology involving C1 segment prolapse at the lateral commissure combined with atrial functional MR represents a challenging scenario for transcatheter edge-to-edge repair (TEER).^10^ Importantly, preprocedural TEE revealed a mitral valve area of only 4.09 cm^2^, which would generally be sufficient in isolated lesions; however, in this patient, the need for multiple clips implantation raised a substantial risk of postprocedural transmitral gradient elevation. Given the patient’s advanced age, multiple comorbidities, and high surgical risk, it seems that the patient could not well tolerate surgical repair. Thus, the central dilemma in managing the case involves a delicate balance between achieving effective regurgitation control and preventing iatrogenic mitral stenosis during TEER.

Before procedure, our multidisciplinary heart team performed detailed planning of the TEER strategy. The first clip was planned to be placed at the lateral commissural region to reduce regurgitation caused by the prolapse segment. A short-wide clip was prioritized for it offers a broader grasping area, facilitating stable capture of the prolapsing segment and effective reduction of the regurgitant orifice. However, following implantation of the first clip, the residual mitral valve area was reduced to 3.09 cm^2^, while significant atrial functional regurgitation persisted, posing a challenge for subsequent intervention. We attempted to use a second short-wide clip to address the residual regurgitation, but observed an elevated transmitral gradient after deployment. A short-narrow clip was then selected to provide targeted coaptation but the gradient still remaining 7 mmHg. The unique ‘Stepless self-locking’ feature of the DragonFly^TM^ system was instrumental in optimizing the positioning of the second clip,^8^ overcoming the conventional ‘all-or-none’ limitation of clip closure. Unlike the MitraClip^TM^ G4, which relies on restricting the relative position of a roughened mandril, or the PASCAL system, which utilizes a heat-set shape-memory design with a single predefined clamping angle,^11^ the DragonFly^TM^ system incorporates specialized grooves on its mandril. This modification enables the device to lock securely at any intermediate angle.^12^ Ultimately, the clip was deployed at a large opening angle, achieving stable leaflet capture with only trace residual regurgitation and a mean transmitral gradient of 5 mmHg.

Achieving an optimal balance between residual mitral regurgitation and transmitral gradient is a key determinant of procedural success in TEER.^13^ More aggressive leaflet approximation reduces regurgitation but at the expense of increased transmitral gradient, whereas a conservative approach preserves valve area but may result in residual regurgitation. This trade-off is particularly pronounced in patients with a small baseline MVA or a mixed regurgitation. Therefore, stepwise device deployment is essential to guide intra-procedural decision-making. In this context, strategies that allow finer control of leaflet approximation, rather than binary full-closure deployment, may provide a more effective means of balancing these competing objectives and optimizing overall procedural outcomes.

Clinical stability and sustained trace regurgitation at the 1-month follow-up validate the safety and efficacy of this tailored strategy. Meticulous intraoperative monitoring of gradients after each device implantation is critical to ensure that the haemodynamic benefits of regurgitation reduction are not offset by iatrogenic stenosis.^14^ Follow-up echocardiography demonstrated stable leaflet position with no evidence of single leaflet device attachment (SLDA), leaflet tear, or subclinical slippage, supporting the short-term safety of the ‘Stepless Self-locking’ strategy. Nevertheless, given that the second clip was deployed in a partially closed position, theoretical risks such as SLDA or clip embolization remain. In addition, follow-up in the present case was now limited to 1 month. Long-term follow-up is highly required to evaluate the durability and stability of the partially closed clip strategy. Moreover, broader applicability of the ‘Stepless Self-locking’ strategy require more validation through studies with larger samples.^15^ Altogether, this case highlights a stepwise, tailored ‘Stepless Self-locking’ strategy that balances effective mitral regurgitation reduction with preservation of valve patency, offering practical insights for the intraoperative decision-making in similarly complex anatomical scenarios.

## Consent of participate and publish

Written informed consent for publication of this case report and images was obtained from the patient in accordance with COPE guidelines.

## Supplementary Material

ytag435_Supplementary_Data

## Data Availability

The original contributions presented in the study are included in the article. Further inquiries can be directed to the corresponding author.

## Lead author biography

Prof. Xianbao Liu is an experienced interventional cardiologist from the Second Affiliated Hospital of Zhejiang University, School of Medicine, Hangzhou, China. His team performed nearly 600 interventional cases of valvular heart diseases every year.

## References

[1] Feldman T, Foster E, Glower DD, Kar S, Rinaldi MJ, Fail PS, et al Percutaneous repair or surgery for mitral regurgitation. N Engl J Med 2011;364:1395–1406. 21463154 10.1056/NEJMoa1009355

[2] Praz F, Borger MA, Lanz J, Marin-Cuartas M, Abreu A, Adamo M, et al 2025 ESC/EACTS guidelines for the management of valvular heart disease. Eur Heart J 2025;46:4635–4736. 40878295 10.1093/eurheartj/ehaf194

[3] Stone GW, Lindenfeld J, Abraham WT, Kar S, Lim DS, Mishell JM, et al Transcatheter mitral-valve repair in patients with heart failure. N Engl J Med 2018;379:2307–2318. 30280640 10.1056/NEJMoa1806640

[4] Hausleiter J, Lim DS, Gillam LD, Zahr F, Chadderdon S, Rassi AN, et al Transcatheter edge-to-edge repair in patients with anatomically Complex degenerative mitral regurgitation. J Am Coll Cardiol 2023;81:431–442. 36725171 10.1016/j.jacc.2022.11.034

[5] Rogers JH, Asch F, Sorajja P, Mahoney P, Price MJ, Maisano F, et al Expanding the Spectrum of TEER suitability: evidence from the EXPAND G4 post approval study. JACC Cardiovasc Interv 2023;16:1474–1485. 37380229 10.1016/j.jcin.2023.05.014

[6] Schnitzler K, Hell M, Geyer M, Kreidel F, Münzel T, von Bardeleben RS. Complications following MitraClip implantation. Curr Cardiol Rep 2021;23:131. 34387748 10.1007/s11886-021-01553-9 PMC8363549

[7] Liu X, Chen M, Han Y, Pu Z, Lin X, Feng Y, et al First-in-human study of the novel transcatheter mitral valve repair system for mitral regurgitation. JACC Asia 2022;2:390–394. 36338402 10.1016/j.jacasi.2022.03.010 PMC9627920

[8] Lin X, Hu W, Ren K, Pu Z, Wang L, Hu P, et al TEER for small mitral valve area: clip deployment at a specific angle. JACC Case Rep 2025;30:104019. 40379369 10.1016/j.jaccas.2025.104019 PMC12145045

[9] Rodés-Cabau J, Mengi S, Salaun E, Paradis JM, Abraham WT. Transcatheter edge-to-edge repair in secondary mitral regurgitation. EuroIntervention 2025;21:e1458–e1478. 41489737 10.4244/EIJ-D-25-00116 PMC12684745

[10] Hausleiter SCM, Weckbach LT, Stocker TJ, Doldi PM, Gmeiner J, Novotny J, et al Impact of mitral valve complexity on outcomes following transcatheter mitral valve edge-to-edge repair. Eur Heart J Cardiovasc Imaging 2026;27:1047–1058. 41609859 10.1093/ehjci/jeag035

[11] Hausleiter J, Stocker TJ, Adamo M, Karam N, Swaans MJ, Praz F. Mitral valve transcatheter edge-to-edge repair. EuroIntervention 2023;18:957–976. 36688459 10.4244/EIJ-D-22-00725 PMC9869401

[12] Zhang T, Zhang W, Zhen X, Luo R, Yang L, Zhang X, et al A transcatheter mitral valve clip with a central filler for mitral valve regurgitation. Biomaterials 2025;321:123317. 40187096 10.1016/j.biomaterials.2025.123317

[13] Tsunamoto H, Yamamoto M, Kagase A, Tokuda T, Sugiura A, Shimura T, et al Using transmitral pressure gradients and residual mitral regurgitation to optimize outcome after transcatheter edge-to-edge repair. J Am Coll Cardiol 2025;86:1684–1700. 41193089 10.1016/j.jacc.2025.07.041

[14] Du Y, Han H, Zhang T, Shen H, Han W, Jia S, et al Prognosis of elevated mitral valve pressure gradient after transcatheter edge-to-edge repair: systematic review and meta-analysis. Curr Probl Cardiol 2024;49:102095. 37778430 10.1016/j.cpcardiol.2023.102095

[15] Sugiura A, Kavsur R, Spieker M, Iliadis C, Goto T, Öztürk C, et al Recurrent mitral regurgitation after MitraClip: predictive factors, morphology, and clinical implication. Circ Cardiovasc Interv 2022;15:e010895. 35193380 10.1161/CIRCINTERVENTIONS.121.010895

