# Supplementary figures and images for: Transcatheter edge-to-edge repair with DragonFlyTM system and ‘stepless self-locking’ strategy in a patient with mixed mitral regurgitation: a case report
Source: Eur Heart J Case Rep. 2026 Jun 20;10(7):ytag435. doi: 10.1093/ehjcr/ytag435 (PMC13345365; doi:10.1093/ehjcr/ytag435)

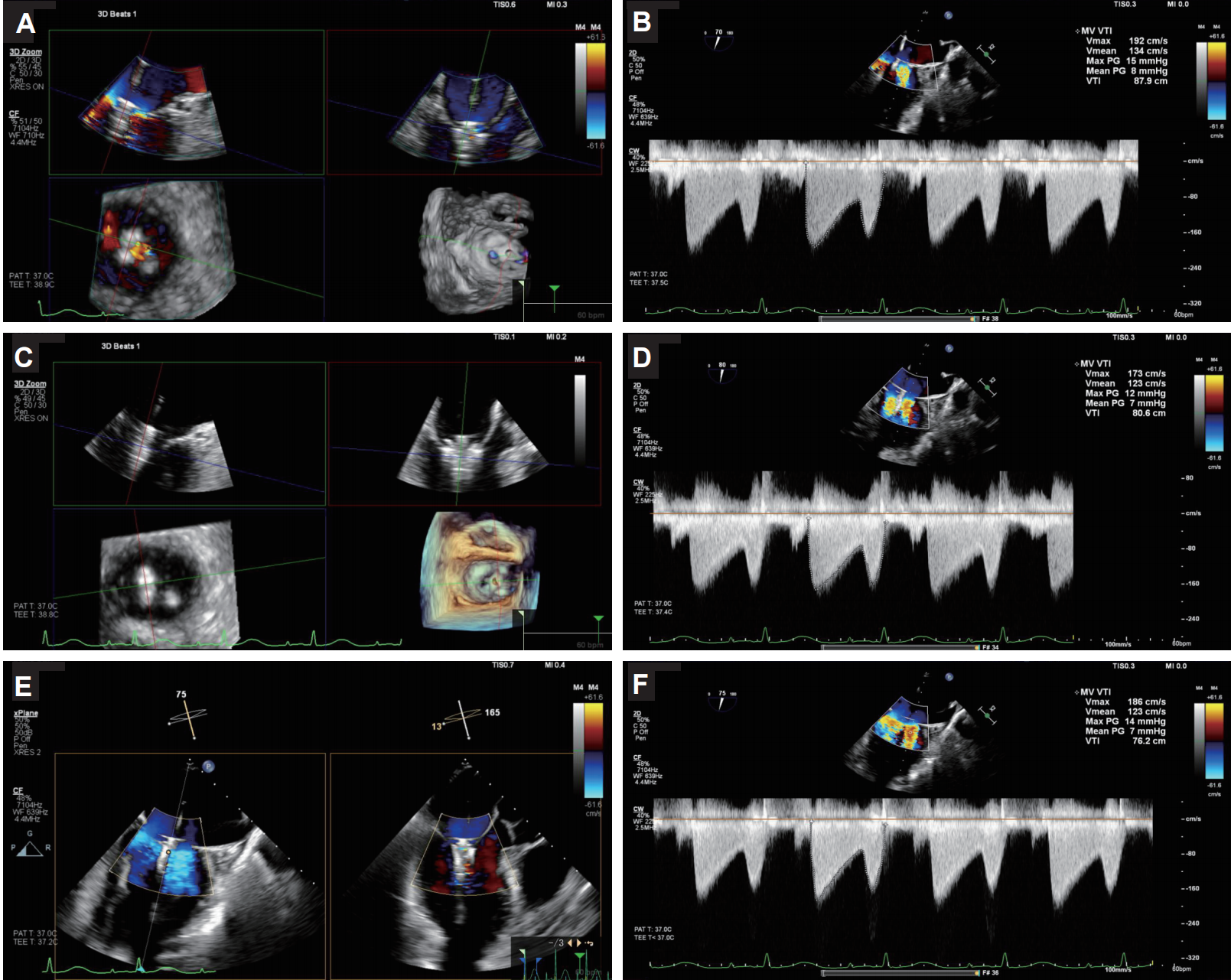

Supplement: ytag435_Supplementary_Data [file ytag435_supplementary_data.zip › S Figure 1.png]
